# Discovery of the Novel Inhibitor Against New Delhi Metallo-β-Lactamase Based on Virtual Screening and Molecular Modelling

**DOI:** 10.3390/ijms21103567

**Published:** 2020-05-18

**Authors:** Xiyan Wang, Yanan Yang, Yawen Gao, Xiaodi Niu

**Affiliations:** Department of Food Science and Engineering, College of Food Science and Engineering, Jilin University, Changchun 130062, China; wangxy17@mails.jlu.edu.cn (X.W.); ynyang18@mails.jlu.edu.cn (Y.Y.); yaweng19@mails.jlu.edu.cn (Y.G.)

**Keywords:** NDM-1, drug-resistant inhibitor, molecular dynamics simulation, virtual screening

## Abstract

New Delhi metallo-β-lactamase (NDM-1), one of the metallo-β-lactamases (MBLs), leads to antibiotic resistance in clinical treatments due to the strong ability of hydrolysis to almost all kinds of β-lactam antibiotics. Therefore, there is the urgent need for the research and development of the novel drug-resistant inhibitors targeting NDM-1. In this study, ZINC05683641 was screened as potential NDM-1 inhibitor by virtual screening and the inhibitor mechanism of this compound was explored based on molecular dynamics simulation. The nitrocefin assay showed that the IC_50_ value of ZINC05683641 was 13.59 ± 0.52 μM, indicating that the hydrolytic activity of NDM-1 can be obviously suppressed by ZINC05683641. Further, the binding mode of ZINC05683641 with NDM-1 was obtained by molecular modeling, binding free energy calculation, mutagenesis assays and fluorescence-quenching assays. As results, ILE-35, MET-67, VAL-73, TRP-93, CYS-208, ASN-220 and HIS-250 played the key roles in the binding of NDM-1 with ZINC05683641. Interestingly, these key residues were exactly located in the catalytic activity region of NDM-1, implying that the inhibitor mechanism of ZINC05683641 against NDM-1 was the competitive inhibition. These findings will provide an available approach to research and develop new drug against NDM-1 and treatment for bacterial resistance.

## 1. Introduction

During the struggle between human and pathogens, β-lactam antibiotics have long become the most powerful weapon for the people against bacterial infections [1]. These β-lactam antibiotic compounds can exactly block the cross-linking of peptidoglycan chains during cell wall biosynthesis of bacterial due to the four-membered β-lactam ring in the structural core of β-lactam antibiotics [2]. However, the antibiotics misuse lead to the extensively antibiotics resistance in pathogenic bacteria. Among most mechanisms of drug-resistance in bacteria strains, the hydrolysis of β-lactam antibiotics by β-lactamase enzymes have been prevalent threat human health [3,4,5]. The hydrolysis mechanism showed that the amide bond in the β-lactam ring of antitiotics can be efficiently hydrolyzed fracture, leading to the loss of antibacterial activity.

Based on the amino acid sequence homology, the β-lactamase enzymes were classified four groups, including class A, C and D as serine β-lactamases (SBLs) and class B as metallo-β-lactamases (MBLs) [6,7]. SBLs can hydrolysis β-lactam ring of antibiotics based on the serine residue around active site via an enzyme-acyl intermediate. This hydrolysis process extends to the formation and fracture of chemical bond. While MBLs can catalyze hydrolysis of β-lactam ring by using the one or two Zn ions in active site [8,9], which realized hydrolysis reaction without proceeding via a covalent intermediate. Due to their unique action mechanism, MBLs can hydrolysis almost all kinds of β-lactam antibiotics such as penicillins, cephalosporins, cephamycins [10]. The research and development of the novel inhibitor targeting MBLs play the crucial role towards the treatment of resistant bacteria [11].

MBLs include three subclasses: B1, B2 and B3 based on their difference on the number of metal ions and amino acid sequences [10]. New Delhi metallo-β-lactamase-1 (NDM-1), Verona integron-encoded (VIM-2), and imipenemase (IMP-1) as B1 subclass were most concerned in antibiotics resistance. Of these, NDM-1 is the most recent additions to the class of MBLs and worrying, since it’s easy to spread on a plasmid and wildly spreaded through bacterial populations, that leading the resistance in pathogenic bacteria [12,13]. More seriously, NDM-1 resisted nearly all available drugs including the inhibitor of SBLs [14,15,16,17]. They can inactivate the last-generation carbapenems with unprecedented efficiency, such as imipenem, meropenem, which regarded as “antibiotics of the last resort”. 

Therefore, screening and design of the effective inhibitors against NDM-1 have attracted widely interestingness of researchers by using kinetic [12], spectroscopic [18], crystallographic [19,20], computational studies [21], and combined investigations techniques. In previous literature, several inhibitors targeting NDM-1 were reported. In 2014, a fungal natural product, aspergillomarasmine A (AMA), was screened as the NDM-1 inhibitors with IC_50_ = 4.0 μM, and it was also showed inhibit activity against VIM-2 [19]. For another example, hypertension drug captopril both D-and L-captopril and their analogues were verified as effective inhibitors for NDM-1 [22] via unique binding mode and competitive inhibition mechanism. In 2018, Yang Xiang et. al. [23] reported that a series of rhodanines was constructed as the broad-spectrum MBLs inhibitors with IC_50_ < 16 μM. These inhibitors can obviously increase the antimicrobial effect of cefazolin. In the same year, the novel ebselen-based dual covalent inhibitors were discovered by Cheng Chen et al. [24]. The correlation analysis shows that the inhibitors could bind with NDM-1 by forming a S-Se bond and the amide bond with Cys221 and Lys224, leading to the bioactivity loss of NDM-1. Based on the virtual screening and experimental testing, Joon S. Kang et. al. also discovered several broad-spectrum MBLs inhibitors [25]. Among 32 compounds selected from the compound library, the most potent inhibitors could efficiently suppress the hydrolysis of NDM-1, IMP-1, and VIM-2 with the IC_50_ of 19 μM, 14 μM, 50 μM respectively. Moreover, 2,6-dipicolinic acid (DPA) [26] and cyclic boronate [27] were also reported as potent inhibitors during the past decade. However, the inhibitors targeting NDM-1 have not been widely used in clinical practice up till now. It means that there is still need for the development and research of the effective and novel inhibitors against NDM-1.

Computational biology is presented as virtual screening, molecular docking, molecular dynamics simulation, and calculation of free energies of binding for small molecule ligands to target proteins [28]. Then, computational biology has become one of the primary methods for pharmaceutical chemistry, which leads to the new compound discovery for the development of pharmaceuticals against bacterial resistance. Among computational biology methods, virtual screening is an efficient approach for the discovery of activity compound from the large library as to the different drug targets, which resulted in that the experimental work could be reduced vastly [29,30]. AutoDock Vina [31] is an academic free software, with several advantages, such as the fast computing speed, high-precision, and easy operation, etc. Then, this software has been extensively used in molecular docking and virtual screening [32,33]. 

In this study, the virtual screening was carried out to search for the novel compounds against NDM-1 from a 182665-compounds library of ZINC database by using AutoDock vina software. Among 20 compounds selected from the compound library, ZINC05683641 was identified as new inhibitors for NDM-1 through experimental test, and the interaction mechanism between the inhibitor and NDM-1 was investigated by using molecular modeling, binding free energy calculations and mutagenesis assays. These findings could provide new strategy against NDM-1 and pathogens infections for future drug design development.

## 2. Results and Discussion

### 2.1. Virtual Screening Analysis

To screen the potential and effective inhibitors against NDM-1, the large ligand library was obtained from ZINC database, which as the ligand during docking process. While the 3D structure of NDM-1 was converted to atom type (.pdbqt) as the receptor in this program. After docking the NDM-1 to everyone of ligand library one by one, the binding affinity (kcal/mol) of ligand-NDM-1 system was obtained. According to the convention of virtual screening [29,34,35], the docking results are usually used as the cutoff to select the active compound. In this research, the binding energies of ligands with protein originated from Autodock vina software were the main results of the virtual screening process. The ligands have the stronger binding affinity with NDM-1 indicating the stronger interaction between ligand and NDM-1, which was more potentially becoming the inhibitors against NDM-1. 

**Figure 1:**
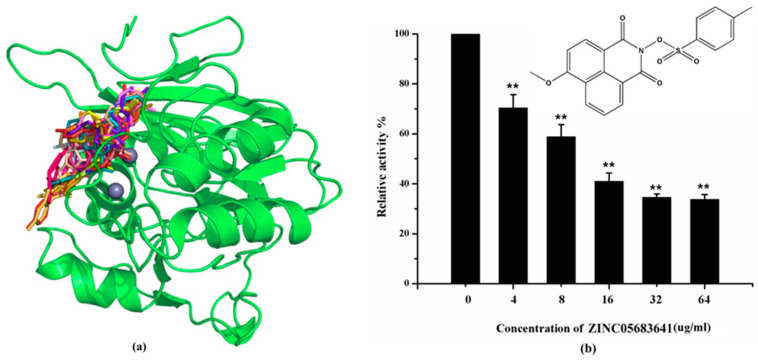
Virtual screening and Enzyme inhibition assay. (**a**) The potential bind mode of 18 compounds to NDM-1 (the Zn ions in active site are shown as spheres); (**b**) The inhibitory effect of ZINC05683641 on NDM-1 and the chemical structure of ZINC05683641. The hydrolysis of nitrocefin in NDM-1 was reduced by the addition of various concentrations on ZINC05683641 after measured at OD492nm. The column diagrams showed the average values for the assays in triplicate, and bars represent the standard deviation. ** indicates *p* < 0.01 compared with the drug-free group; two-tailed Student’s test.

We selected the top 100 hits in the following study according to the binding affinity and all the binding affinity of 100 hits was less than −8.0 kcal/mol. Above100 hits after pre-filtered with high binding affinity were subjected to physicochemically profiled using SwissADME [36]. Out of the 100 compounds, 88 of them passed Lipinski’s Rule-of-Five [37] and Veber’s rule [38]. Then, compounds with high binding affinity and complied with the Lipinski’s Rule-of-Five and Veber’s rule were used to further screening by investigating the binding site with NDM-1 receptor. As known, the active site of NDM-1 was a hydrophobic channel surrounding the two Zn ions reported in previous literature [26]. The binding sites of the 88 ligands bind to the catalytic active region of NDM-1 were analyzed by PyMOL and ligplot tools. The finally 18 compounds were bound to active site of NDM-1 more closely shown in Figure 1a, and the binding affinity of them were less than −8.8 kcal/mol (Table 1).

### 2.2. Nitrocefin Assay

The inhibitory activities of 18 compounds against NDM-1 were tested by nitrocefin assay. The purified NDM-1 was incubated with drugs and nitrocefin, then the absorbance values of OD_492nm_ were determined as the nitrocefin hydrolysis. Only compounds ZINC05683641 exhibited larger inhibition rates than 50% at a concentration of 16 μM (Figure 1b). In addition, the half-maximal inhibitory concentration (IC_50_) value of ZINC05683641 were determined as 13.59 ± 0.52 μM, implying that ZINC05683641 is the potential novel inhibitor against NDM-1. On the other hand, as shown in Table 1, the IC_50_ values of other compounds against NDM-1 were all more than 200 μM, which means that these compounds almost have none inhibitory to NDM-1. Therefore, ZINC05683641 was identified the potential inhibitor to NDM-1 according to virtual screening and nitrocefin assay. Subsequently, the stable binding mode of ZINC05683641 with NDM-1 and the interaction mechanism between ligand and protein at the atomic level were explored based on molecular modelling, and the chemical structure of ZINC05683641 was shown in Figure 1b. 

### 2.3. Antibacterial Activity Assay

It was found that ZINC05683641 can improve the antibacterial effect of meropenem. The antibacterial activity of ZINC05683641 alone and the combination of ZINC05683641 with meropenem against *E. coli* BL21 (pET28a-SP-NDM-1) were determined by the minimum inhibitory concentrations (MICs). As shown in Table 2, the inhibitor alone (512 μg/mL) did not inhibit cell growth, revealing that the inhibitor had little effect on *E. coli* BL21 (pET28a-SP-NDM-1) cells alone. While, the MICs of meropenem reduced fourfold due to addition of ZINC05683641 with concentrations of 64 and 128 μg/mL against NDM-1-positive strains. These results revealing that ZINC05683641 rescued the antibacterial activity of meropenem whose against NDM-producing isolates. Moreover, FICI calculation results also showed synergistic effects of ZINC05683641 with meropenem (FICI = 0.375 and 0.500, FICI ≤ 0.5 denotes synergy [39]).

### 2.4. The Potential Binding Mode of Compound ZINC05683641 with NDM-1

**Figure 2:**
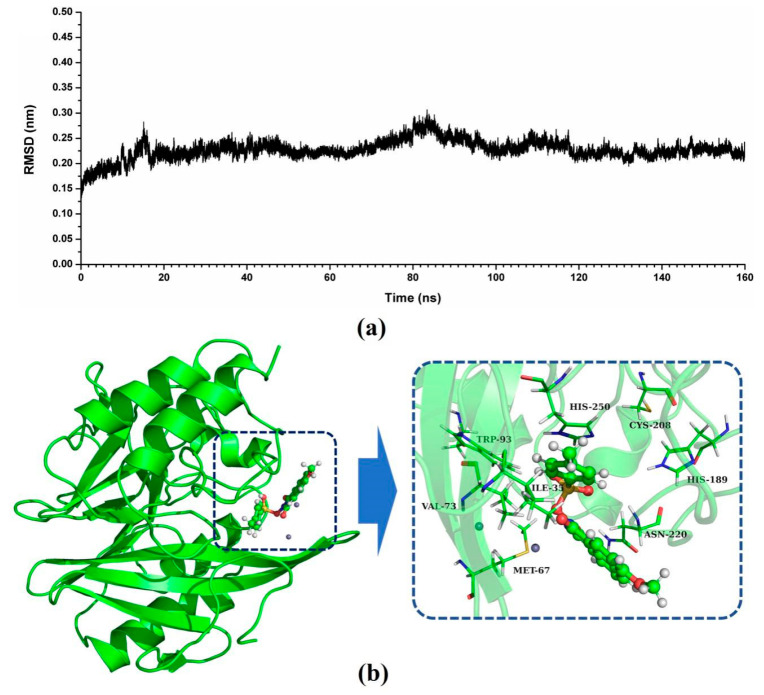
Identification of the binding sites of ZINC05683641 with NDM-1. (**a**) The RMSD values of NDM-1 with ZINC05683641 complex via simulation times; (**b**) The potential binding mode of ZINC05683641with NDM-1 based on MD simulation.

In this study, the potential binding mode of NDM-1-ZINC05683641 complex were identified by molecular docking, molecular modelling, and binding free energy calculations. According to the result of virtual screening and nitrocefin assay, the compound ZINC05683641 could bind to the active site of NDM-1 and effectively inhibit the hydrolytic activity of NDM-1. Then, 3D structure of NDM-1-ZINC05683641 complex obtained by molecular docking was used as the initial coordinates for the molecular dynamics simulation. The stable structure of the complexes was obtained through the 160-ns standard molecular modelling. After 160 ns molecular dynamics simulation, it was shown that the complex system reached the equilibrium by the analysis of the root means square deviation (RMSD), as shown in Figure 2a. The NDM-1-ZINC05683641 complex system equilibrated at 100 ns with the RMSD values nearly to 0.2 nm, which indicates each structure almost reach stability after 100 ns, and the final 60 ns MD simulation was used to further analysis.

As shown in Figure 2b, a stable structure of NDM-1-ZINC05683641 complex was obtained after MD simulation. The inhibitor ZINC05683641 was bound to the active site consisting of two Zn ions in the hydrophobic pocket. Based on the 3D structure of NDM-1-ZINC05683641 complex, it was obviously shown that the imidazole ring of the side chain of HIS-250 is parallel to the plane of the benzene ring of ZINC05683641, implying that a strong π–π interaction between ZINC05683641 and His250 exists. At the same time, on the other side of benzene ring of ligand, there is the strong hydrophobic interaction between the isobutane chain of ILE-35 and the benzene ring of ZINC05683641. In addition, the propane chain of VAL-73 also can form the hydrophobic interaction with the benzene ring. Thus, the benzene ring end of ligand was anchored in the active site of NDM-1 via the special sandwich structure: (HIS-250)/(ZINC05683641)/(ILE-35 and VAL-73). While, on the end of the 2-methyl-1H-benzo[*de*]isoquinoline-1,3(2H)-dione group of ZINC05683641, the binding mechanism is simpler. Figure 2b showed that the side chain of ASN-220 can form the strong interaction with the other side of ligand relative to benzene ring. Furthermore, the residues MET-67, TRP-93, HIS-189, and CYS-208 appear to be crucial in maintaining the binding stability of ZINC05683641 to NDM-1.

### 2.5. Confirmation of the Binding Mode in the Complex

**Figure 3:**
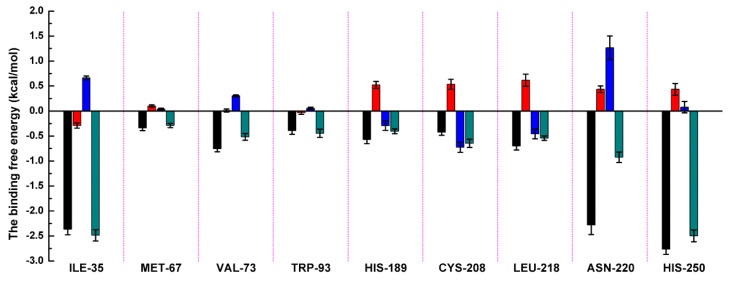
Decomposition of the binding energy on a per-residue basis in the NDM-1-ZINC05683641 by the MM-GBSA method. The histogram chart shows the van der Waals (black), electrostatic (red), solvation (blue), and total (green) contributions for the complexes.

Based on the above results, it was shown that residues of ILE-35, MET-67, VAL-73, TRP-93, His189, Cys208, ASN-220, and HIS-250 play the crucial role in the binding of ZINC05683641 with NDM-1. To verify this hypothesis, the binding free energy between ligand and protein and the decomposition free energy of each residue contributing to the binding were calculated by using the molecular mechanics generalized born surface area (MM-GBSA) method. As shown in Figure 3, most of the decomposed energy interaction originated from van der Waals contribution, apparently through hydrophobic interactions, while the electrostatic interactions appeared to be a minor influence on those key residues in complex system.

Residues of ILE-35, ASN-220 and HIS-250 showed the strongest binding free energy contributed to the binding with ZINC05683641, with the *ΔE_total_* values of −2.49, −0.93 and −2.50 kcal/mol, respectively. Notably, these three residues also have the strongest van der Waals interaction with ZINC05683641, with the *ΔE_vdw_* values of −2.37, −2.28 and −2.77 kcal/mol, respectively. These results confirmed that van der Waals interaction in the mainly contribution between the side chains of ILE-35, ASN-220 and HIS-250 with ZINC05683641 at binding site. The 3D structure of the complex system shown that ILE-35 has a close distance with the benzene ring of ZINC05683641, leading to the strong hydrophobic interaction between ligand and protein (with the *ΔE_vdw_* values of −2.37 kcal/mol). Similarly, VAL-73 also have the stronger van der Waals interaction (with a *ΔE_vdw_* of −0.75 kcal/mol) with ligand via the close distance of side chain with benzene ring of ZINC05683641, leading to the strong total binding free energy with inhibitor (with a *ΔE_total_* of −0.52 kcal/mol).

In addition, due to the close distance between the side chain of ASN-220 and aromatic plane of inhibitor, the strong interaction exists (with the *ΔE_vdw_* values of −2.28 kcal/mol). While, for HIS-250, the strong π–π interaction between imidazole ring of HIS-250 and benzene ring of inhibitor results in the strongest van der Waals contribution in this complex system (with the *ΔE_vdw_* values of −2.77 kcal/mol), and the benzene ring moiety of ZINC05683641 become the major contribution to the binding of ligand with protein. Then, we believed that due to the strongest van der Waals contribution, HIS-250 is the essential key residue in this complex system.

**Figure 4:**
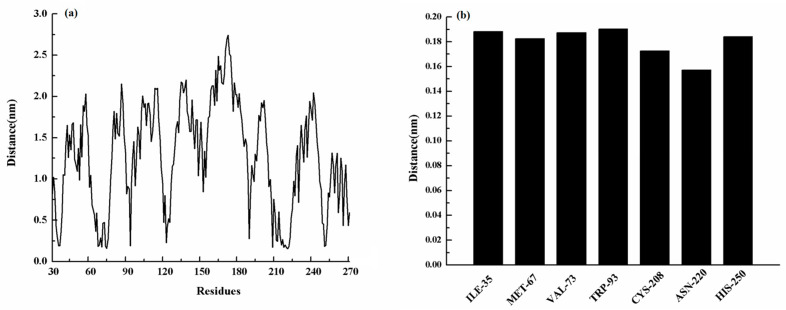
(**a**) The distance between the whole residues of NDM-1 and ZINC05683641; (**b**) the distances between residues of the binding sites of NDM-1 and ZINC05683641.

Moreover, MET-67, TRP-93, HIS-189, and CYS-208 also have stronger binding free energy with ZINC05683641 except for ILE-35, ASN-220 and HIS-250, with *ΔE_total_* values of −0.29, −0.45, −0.41, and −0.65 kcal/mol, respectively. As shown in Figure 3, only the van der Waals interaction has the major contribution to the binding of ligand with protein, and electrostatic, solvation interactions only have minor influence on the complex even the negative effect such as HIS-189. Due to the strong unfavorable electrostatic contribution at 0.52 kcal/mol on the residue HIS-189 bound to inhibitor result in the weaker binding energy with *ΔE_total_* value of −0.41 kcal/mol than others on the complex. Based on the above results, most of binding energy between NDM-1 with ZINC05683641 was contributed by van der Waals interaction, and residues of ILE-35, MET-67, VAL-73, TRP-93, CYS-208, ASN-220 and HIS-250 contributed to the major binding energy in this complex system, which is good consistent with the above results (Figure 2b). Moreover, the average distances between each residue of NDM-1 and ZINC05683641 during MD simulations were calculated. As shown in Figure 4, the distances between ZINC05683641 and the residues ILE-35, MET-67, VAL-73, TRP-93, CYS-208, ASN-220 and HIS-250 were less than 0.2 nm. It is confirmed that the strong interactions existed between the above residues and ligand, and these residues are the key residues in NDM-1-ZINC05683641 complex.

From previous literature [40,41], some interaction mechanisms in kinds of substrates (such as nitrocefin, ampicillin, meropenem, imipenem etc.) hydrolyzed by NDM-1 were reported by structure-based computational methods in recent years. For example, residues ILE-35, TRP-93, HIS-189, CYS-208, LYS-211, GLY-219, ASN-220 and HIS-250 are the key residues in nitrocefin binding to NDM-1; residues TRP-93, ASP-124, CYS-208, GLY-219, ASN-220 and HIS-250 in NDM-1-ampicillin complex; ASP-124, HIS-120, LYS-211, HIS-122, HIS-250, HIS-189, LYS-211, VAL-73 and TRP-93 in NDM-1- meropenem complex. While these antibiotics bound to the catalytic activity region of NDM-1 results in hydrolysis by the lactamase. According to these results, the residues which played essential roles in the binding of ZINC05683641 to NDM-1 were just located in the catalytic active region of NDM-1 hydrolyzing substrates. Therefore, we believed that ZINC05683641 could inhibit the hydrolysis activity of NDM-1 effect via the competitive inhibition.

### 2.6. Identification of the Competitive Inhibition Mechanism

To verify this inhibition mechanism, residues of TRP-93 and HIS-250 were mutated to alanine, and the similar MD simulation was carried out for the mutants (W93A and H250A) with ZINC05683641 complexes. Subsequently, the total binding free energy of wild-type NDM-1 (WT-NDM-1) and NDM-1 mutants with inhibitor were calculated by using MM-GBSA method. It can be seen from Table 3, the *ΔG_bind_* values of WT-NDM-1-inhibitor, W93A-inhibitor and H250A-inhibitor complexes were −26.96, −25.00, −22.30 kcal/mol, respectively. The binding free energy of NDM-1-ZINC05683641 complexes were decreased in the following sequence: WT > W93A-NDM-1 > H250A-NDM-1 due to the mutation of residues in the binding sites of NDM-1, which indicates that the binding of WT-NDM-1 with ZINC05683641 is stronger than those of mutants.

Furthermore, this competitive inhibition mechanism was verified by fluorescence quenching method. In previous literature [42,43,44], fluorescence quenching method are extensively used in the interaction mechanism research between ligand and protein. The binding constants of NDM-1 with ZINC05683641 were measured by fluorescence quenching method in this research. As shown in Table 2, the binding constants (*K_A_*) were 4.17 ± 1.02, 3.05 ± 0.83, 2.15 ± 0.88 10^5^ L/mol for WT-NDM-1, W93A-NDM-1, and H250A-NDM-1 with ZINC05683641, respectively, suggesting that the binding affinities of WT-NDM-1 and NDM-1 mutants were also decreased in the following sequence: WT > W93A-NDM-1 > H250A-NDM-1, which is consistent with the results of the binding free energy obtained from the MD simulation. It is believed that the 3D structure of NDM-1-ZINC05683641 complex is reliable by MD simulation, and residues of ILE-35, MET-67, VAL-73, TRP-93, CYS-208, ASN-220 and HIS-250 played crucial roles in the binding of ZINC05683641 to NDM-1.

**Figure 5:**
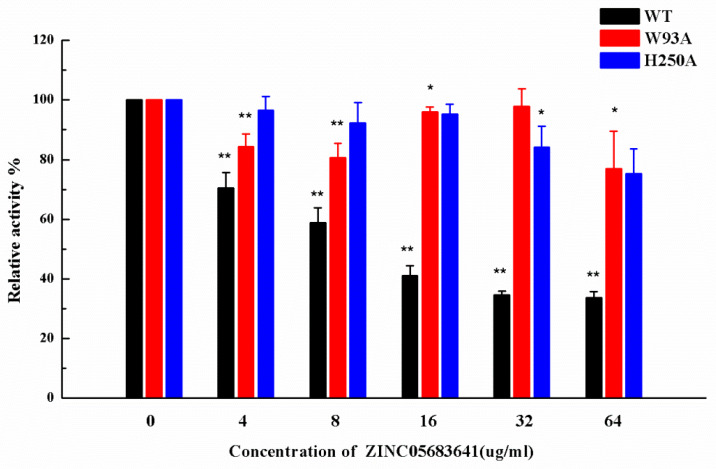
The inhibitory effect of ZINC05683641 on WT-NDM-1 and its mutants. The column diagrams show the hydrolysis activity of WT-NDM-1 (black), W93A (red), and H250A (blue). The hydrolysis activity of nitrocefin by WT-NDM-1 was reduced by addition to ZINC05683641 with the final concentrations of 4, 8, 16, 32 and 64 μg/mL. However, the addition of ZINC05683641 to W93A and H250A did not lead to inhibitory effects. All data are shown as means ± SD of three independent experiments. * indicates *p* < 0.05 and ** indicates *p* < 0.01 compared with the drug-free group; two-tailed Student’s test.

Subsequently, the inhibitory activity of ZINC05683641 against mutants was tested by the nitrocefin assays. Figure 5 shows that the biological activity of mutants has no obviously change compared with WT-NDM-1, implying that the characterization of the mutants is the same as that of WT-NDM-1. But it is amazing, that ZINC05683641 showed almost no inhibition against NDM-1 mutants W93A and H250A. The findings indicate that due to the mutation of residues in the binding sites, the binding affinity of ZINC05683641 with NDM-1 decreased, leading to a loss of inhibitory activity. Therefore, our results indicated that the MD simulation produced a reliable NDM-1-ZINC05683641 complex, and residues of ILE-35, MET-67, VAL-73, TRP-93, CYS-208, ASN-220 and HIS-250 played crucial roles in the binding of ZINC05683641 to NDM-1.

## 3. Method and Computation

The 3D structure of NDM-1 was obtained from the Protein Data Bank (PDB) with the PDB ID: 3ZR9 as the receptor during virtual screening process [22]. The ligand library consisting of 182665 compounds was gained from a commercial subset made by Sigma-Aldrich of ZINC database. A grid box of was created including the two Zn ions in the active site of NDM-1 receptor, and centered on the mass center of the ligand. The virtual screening was performed by AutoDock Vina (The Scripps Research Institute, CA, USA) [28] package to searching for the potential inhibitors against NDM-1. The stable binding modes of inhibitors with NDM-1 were obtained based on the classical molecular modeling by using Gromacs 4.5.5 software (University of Groningen, Groningen, Netherland) [45]. The interaction at the atomic level between new finding inhibitor and NDM-1 was investigated through the calculation of the binding free energy between ligand and receptor by MM-GBSA method [46,47].

Data were described as mean ± SD from three independent experiments, and the significance levels hydrolytic activity essay was evaluated by two-tailed Student’s test using SPSSV13.0 (IBM, Armonk, New York, USA) In details, * indicating *p* < 0.05 and ** indicating *p* < 0.01 compared with the control group, *p* < 0.05 was considered statistically significant.

The computational methods and other details of process are described in the Supplementary Materials.

## 4. Conclusions

In this study, compound ZINC05683641 was discovered as a new available inhibitor against NDM-1 with IC_50_ value of 13.59 ± 0.52 μM through the virtual screening method and enzyme inhibition assay. Besides, the binding mode and interaction mechanism of ZINC05683641 against NDM-1 were explored at the atomic level by using molecular modeling, binding free energy calculation, mutagenesis assays and fluorescence-quenching assays. The results showed that residues of ILE-35, MET-67, VAL-73, TRP-93, CYS-208, ASN-220 and HIS-250 contributed to the major binding energy in the binding of ZINC05683641 with NDM-1, implying that these residues played crucial roles in the NDM-1-ZINC05683641 complex. As the previous literature reported, these residues are just located in the catalytic active region of NDM-1. Therefore, we believed that ZINC05683641 could inhibit the hydrolysis activity of NDM-1 effect via the competitive inhibition. Moreover, the results of enzyme inhibition assays of mutants treated with ZINC05683641 were good agreement with the above results. These findings could contribute to the research and development of novel drug-resistant inhibitor design, and laid function for food-borne pathogens infections.

## Figures and Tables

**Table 1:** Screen 18 ligands during virtual screening and inhibition ratio.

Ligands	Structure	Binding Affinity (kcal/mol)	IC_50_(μM)
ZINC00639379	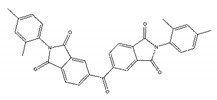	−10.0	207.28 ± 15.51
ZINC03198432	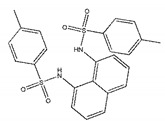	−9.4	>300
ZINC25558269	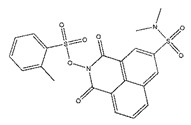	−9.2	N/A ^a^
ZINC02938448	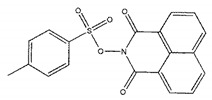	−9.2	>300
ZINC02079077	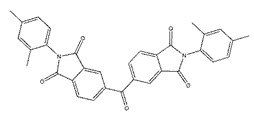	−9.2	107.68 ± 5.64
ZINC12524283	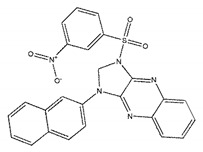	−9.1	>300
ZINC02953923	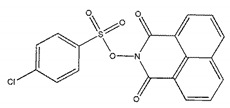	−9.1	>300
ZINC06166781	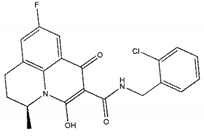	−9.1	>300
ZINC25558273	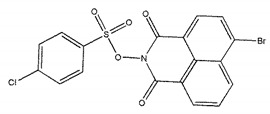	−9.0	>300
ZINC11865709	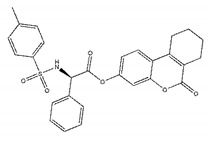	−9.0	>300
ZINC05683641	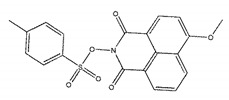	−9.0	13.59 ± 0.52
ZINC02577071	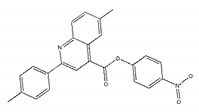	−9.0	>300
ZINC06421319	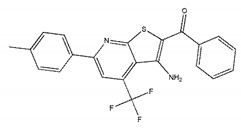	−8.9	N/A
ZINC04218138	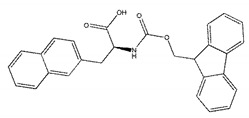	−8.9	N/A
ZINC20115378	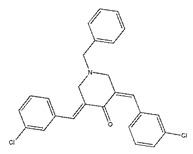	−8.8	>300
ZINC25558691	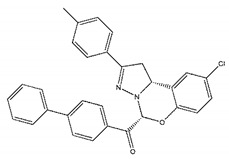	−8.8	N/A
ZINC25694564	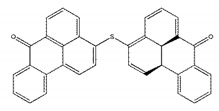	−8.8	N/A
ZINC01799114	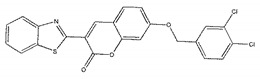	−8.8	>300

^a^ Not available but showing no inhibitory activity at a concentration of 1 mM. IC_50_ values of these compounds were shown as mean ± standard deviation (SD), *n* = 3.

**Table 2:** MIC and FICI of meropenem in combination with ZINC05683641 against *E. coli* BL21 (pET28a-SP-NDM-1).

	Meropenem	ZINC05683641	Meropenem (+64 μg/mL ZINC05683641)	Meropenem (+128 μg/mL ZINC05683641)
MIC (μg/mL)	32	>512	8	8
FICI	-	-	0.375	0.50

**Table 3:** The binding free energy (kcal/mol) of WT, W93A, and H250A with ZINC05683641 complex and the values of the binding constants (KA) (1 × 10^5^ L mol^−1^) based on the fluorescence spectroscopy quenching.

Energy Components (kcal/mol)	WT	W93A	H250A
*ΔE_ele_*	−12.12 ± 0.96	−14.11 ± 0.75	−7.23 ± 1.12
*ΔE_vdw_*	−38.05 ± 1.15	−35.43 ± 0.54	−34.34 ± 1.18
*ΔE_MM_*	−50.17 ± 1.20	−49.54 ± 1.12	−41.57 ± 1.34
*ΔG_sol_*	23.21 ± 0.92	24.53 ± 0.85	19.27 ± 1.13
*ΔG_bind_*	−26.96 ± 0.88	−25.00 ± 0.56	−22.30 ± 0.99
*K_A_*	4.17 ± 1.02	3.05 ± 0.83 *	2.15 ± 0.88 *

All data are shown as means ± SD, *n* = 3. * indicates *p* < 0.05 compared with the WT-NDM-1 group; two-tailed Student’s test.

## Supplementary Materials

Supplementary materials can be found at https://www.mdpi.com/1422-0067/21/10/3567/s1. Method and computation.
