# Supplementary material for: Discovery of the Novel Inhibitor Against New Delhi Metallo-β-Lactamase Based on Virtual Screening and Molecular Modelling
Source: Int J Mol Sci. 2020 May 18;21(10):3567. doi: 10.3390/ijms21103567 (PMC7279046; doi:10.3390/ijms21103567)
Supplement: Supplementary file 1 [file ijms-21-03567-s001.pdf]

Method and Computation

Vitual Screening Process

The crystal structure of NDM-1 was obtained from the Protein Data Bank (PDB), and the PDB codes of 3ZR9 were used as initial coordinates for the *Vitual screening* calculations using AutoDock Vina established on Gromacs 4.5.5 package.[1, 2] The Lamarckian genetic algorithm (LGA) was applied for the docking calculations. All torsional bonds of the drug were free to rotate while NDM-1 was held rigid. The polar hydrogen atoms were added for NDM-1 using the AutoDock tools, and the Kollman united atom partial charges [2] were assigned. Energy grid maps for all possible ligand atom types (.PDBQT) were generated by using Autogrid 4 before performing AutoDock Vina. To improved computing efficiency, the AutoDock Vina search space was minimized to cover binding sites using the following dimensions: center_x = 20.47 Å, center_y = -7.55Å, and center_z = 23.22 Å; and size_x = 16 Å, size_y = 18 Å, and size_z = 14 Å.

The clusters were ranked according to the lowest energy representative from each cluster. Three ligand docking poses suggesting preferential binding to the loop region were named Pose 1, Pose 2, Pose 3 and Pose 4. In NDM-1- ZINC05683641 complex, Pose 1 had the lowest energy conformation (–9.0 kcal/mol) and the most populated cluster (31) compared to Pose 2 (–8.8 kcal/mol, 17) , Pose 3 (–8.3 kJ mol^{-1}, 12) and Pose 4 (–7.3 kJ mol^{-1}, 9) . For other systems, the docking poses are similar to the NDM-1- ZINC05683641 system. Therefore, the lowest energy conformation (Pose 1) in the most populated cluster was chosen for further study [2].

Molecular dynamics simulation

All the simulations and the analysis of the trajectories were performed with Gromacs 4.5.1

software [3] using the Amber99sb force field and the TIP3P water model [4]. The NDM-1- drug system was first energy-relaxed with 2000 steps of the steepest-descent energy minimization followed by another 2000 steps of conjugate-gradient energy minimization. The system was then equilibrated by a 500 ps molecular dynamic run with positional restraints on both the protein and the ligand in order to allow relaxation of the solvent molecules. The first equilibration run was followed by a 160 ns MD run without position restraints on the solute. The first 30 ns of the trajectory were not used in the subsequent analysis for minimization of convergence artefacts. Equilibration of the trajectory was checked by monitoring the equilibration of quantities such as the root-mean-square deviation (RMSD) with respect to the initial structure, the internal protein energy, and the fluctuations calculated for different time intervals. The electrostatic term was described with the particle mesh Ewald algorithm. The LINCS [5] algorithm was used to constrain all bond lengths. For the water molecules, the SETTLE algorithm [5] was used. The dielectric permittivity was set as 1 and a time step of 2 fs was used. All atoms were given an initial velocity determined from the Maxwell distribution at the desired initial temperature of 300 K. The density of the system was adjusted during the first equilibration runs at NPT conditions by weak coupling to a bath of constant pressure (P_0 = 1 bar, coupling time $_P$ = 0.5 ps) [6]. In all simulations, the temperature was maintained close to the intended values by weak coupling to an external temperature bath with a coupling constant of 0.1 ps. The proteins and the rest of the system were coupled separately to the temperature bath. The structural cluster analysis was carried out by using the method described by Daura and co-workers with a cutoff of 0.25 nm [6].

The parameters of ZINC05683641 were estimated with the antechamber program [7] and the

AM1-BCC partial atomic charges from the Amber suite [8]. Analysis of the trajectories was performed using the VMD, PyMOL, and Gromacs analysis tools.

Calculation of the binding free energy

In this work, the binding free energies have been calculated using the MM-GBSA approach [9, 10] supplied with the Amber 10 package. We chose a total number of 100 snapshots evenly from the last 10 ns on the MD trajectory with an interval of 10 ps. The MM-GBSA method can be conceptually summarized as follows:

$$G_{bind} = G_{complex} - [G_{protein} + G_{lig}] \quad (1)$$

$$E_{MM} = E_{vdw} + E_{ele} \quad (2)$$

$$G_{sol} = G_{ele,sol} + G_{nonpolar,sol} \quad (3)$$

where E_{MM} is the summation of the van der Waals (E_{vdw}) and the electrostatic (E_{ele}) interaction energies.

In addition, G_{sol}, which denotes the solvation free energy, can be computed as the summation of an electrostatic component ($G_{ele,sol}$) and a nonpolar component ($G_{nonpolar,sol}$), as shown in Equation (3).

The interactions between ligands and each residue in the binding site of NDM-1were analyzed using the MM-GBSA decomposition process applied in the MM-GBSA module in Amber 10. The binding interaction of each ligand-residue pair includes three terms, namely, the Van der Waals contribution (E_{vdw}), the electrostatic contribution (E_{ele}), and the solvation contribution (E_{sol}). All energy components were calculated using the same snapshots as the free energy calculation.

Principal component analysis

Principle component analysis (PCA) enables isolation of the essential subspace from local fluctuations via the calculation of a set of eigenvectors that describe the correlated motions of atoms within the MD simulation [11]. PCA was performed to address the collective motions of the NDM-1-inhibitor complexes using the positional covariance matrix, *C*, of the atomic coordinates and its eigenvectors. PCA was performed with the Gromacs 4.5.1 module and the trajectories were obtained from the previous MD simulations.

Fluorescence-quenching assay

According to Dong et al., Niu et al. and Qiu et al., the binding constants (K_A) of WT-NDM-1 and two mutants NDM-1 with inhibitor were measured using the fluorescence-quenching method [12-14] .

Site-Directed Mutagenesis

The recombinant plasmid pET28a-NDM-1 was constructed according to previous reports [15] encoding WT-NDM-1. Plasmids encoding W93A-NDM-1 or H250A-NDM-1 were constructed by using a QuikChange site-directed mutagenesis kit. The template plasmid was the pET28a-NDM-1 plasmid, as noted above.

Expression and purification of WT-PLY and mutants

Plasmids harboring the coding sequences for WT-NDM-1, W93A-NDM-1, and H250A-NDM-1 were transformed into *E. coli* BL21 (DE3). The *E. coli* strain was cultured in 1,000 mL of LB broth supplemented with 50 μg mL^{-1} kanamycin until the OD 600 nm of the cultures reached 0.6–0.8. Protein expression was induced by using 0.3 mM IPTG for 12 h at 16 °C. The cells were harvested and washed once with sterile PBS. The pellets were suspended in PBS and lysed by sonication. The cell lysate was centrifuged at 12,000*g* for 30 min, and the supernatants were

collected for the subsequent protein purification as described by Liu et.al [16].

Nitrocefin assay

A nitrocefin assay was used for the identification of potentially effective inhibitors and for further determination of the inhibitory effect of ZINC05683641 on the hydrolysis activities of NDM-1. The initial identifying of NDM-1 inhibitory activities was carried out on the compounds at a concentration of 1 mM (Table 1), and the compounds with greater than 50% inhibition at 1 mM were further tested to obtain their IC_{50} values against NDM-1. Nitrocefin serves as an indicator whose colour changes from yellow to red with increased hydrolysis. NDM-1 were incubated with various concentrations of inhibitor (0, 4, 8, 16, 32, 64 μg/mL) in phosphate buffer (pH = 7.4) at room temperature for 20 minutes, and then, 25 μL of nitrocefin was added to the mixture. After 10 minutes of incubation, the samples were read at OD_{492nm} to determine the level of nitrocefin hydrolysis.

Antibacterial activity assays

The MIC values for meropenem alone and combined with ZINC05683641 against *E.coli* BL21 (pET28a-SP-NDM-1) were determined following the guidelines of the Clinical and Laboratory Standards Institute by the broth microdilution method.(CLSI, Wayne, PA, USA, 2016). *E.coli* BL21 containing plasmids pET28a NDM-1 transferred to 2 mL of LB liquid medium and grown at 37 overnight then diluted to 5×10^6 CFUs/ml. The MIC was interpreted as the lowest concentration of the drug that completely inhibited the visible growth of bacteria after incubating plates for 18 h at 37 ℃. Each inhibitor was tested in triplicate in at least two independent experiments and the highest MIC value was reported. The synergistic effects were evaluated by determining the fractional inhibitory concentration index (FICI), which was

interpreted according to the European Committee on Antimicrobial Susceptibility Testing as follows: FICI ⩽ 0.5 denotes synergy, 0.5 < FICI ⩽ 4 denotes no interaction; and FICI > 4 denotes antagonism [17].

Materials

Meropenem, nitrocefin and 20 compounds from virtual screening were purchased from Sigma-Aldrich (St. Louis, MO, USA).

References

1. Hu, R.; Barbault, F.; Maurel, F.; Delamar, M.; Zhang, R., Molecular dynamics simulations of 2-amino-6-arylsulphonylbenzonitriles analogues as HIV inhibitors: interaction modes and binding free energies. *Chem Biol Drug Des* **2010,** 76, (6), 518-26. doi: 10.1111/j.1747-0285.2010.01028.x
2. Morris, G. M.; Goodsell, D. S.; Huey, R.; Olson, A. J., Distributed automated docking of flexible ligands to proteins: parallel applications of AutoDock 2.4. *J Comput Aided Mol Des* **1996,** 10, (4), 293-304.
3. Hess, B.; Kutzner, C.; van der Spoel, D.; Lindahl, E., GROMACS 4: Algorithms for Highly Efficient, Load-Balanced, and Scalable Molecular Simulation. *J Chem Theory Comput* **2008,** 4, (3), 435-47. doi: 10.1021/ct700301q
4. Jorgensen, W. L.; Chandrasekhar, J.; Madura, J. D.; Impey, R. W.; Klein, M. L., Comparison of simple potential functions for simulating liquid water. *The Journal of chemical physics* **1983,** 79, (2), 926-935.
5. Ryckaert, J.-P.; Ciccotti, G.; Berendsen, H. J., Numerical integration of the cartesian equations of motion of a system with constraints: molecular dynamics of n-alkanes. *Journal of Computational Physics* **1977,** 23, (3), 327-341.
6. Berendsen, H. J.; Postma, J. v.; van Gunsteren, W. F.; DiNola, A.; Haak, J., Molecular dynamics with coupling to an external bath. *The Journal of chemical physics* **1984,** 81, (8), 3684-3690.
7. Wang, J.; Wang, W.; Kollman, P. A.; Case, D. A., Automatic atom type and bond type perception in molecular mechanical calculations. *Journal of molecular graphics and modelling* **2006,** 25, (2), 247-260.
8. Jakalian, A.; Jack, D. B.; Bayly, C. I., Fast, efficient generation of high‐quality atomic charges. AM1‐BCC model: II. Parameterization and validation. *Journal of computational chemistry* **2002,** 23, (16), 1623-1641.
9. Punkvang, A.; Saparpakorn, P.; Hannongbua, S.; Wolschann, P.; Beyer, A.; Pungpo, P., Investigating the structural basis of arylamides to improve potency against M. tuberculosis strain through molecular dynamics simulations. *Eur J Med Chem* **2010,** 45, (12), 5585-93. doi: 10.1016/j.ejmech.2010.09.008
10. Schaffner-Barbero, C.; Gil-Redondo, R.; Ruiz-Avila, L. B.; Huecas, S.; Lappchen, T.;

den Blaauwen, T.; Diaz, J. F.; Morreale, A.; Andreu, J. M., Insights into nucleotide recognition by cell division protein FtsZ from a mant-GTP competition assay and molecular dynamics. *Biochemistry* **2010,** 49, (49), 10458-72. doi: 10.1021/bi101577p

11. Amadei, A.; Linssen, A. B.; Berendsen, H. J., Essential dynamics of proteins. *Proteins* **1993,** 17, (4), 412-25. doi: 10.1002/prot.340170408

12. Dong, J.; Qiu, J.; Zhang, Y.; Lu, C.; Dai, X.; Wang, J.; Li, H.; Wang, X.; Tan, W.; Luo, M.; Niu, X.; Deng, X., Oroxylin A inhibits hemolysis via hindering the self-assembly of alpha-hemolysin heptameric transmembrane pore. *PLoS Comput Biol* **2013,** 9, (1), e1002869. doi: 10.1371/journal.pcbi.1002869

13. Niu, X.; Qiu, J.; Wang, X.; Gao, X.; Dong, J.; Wang, J.; Li, H.; Zhang, Y.; Dai, X.; Lu, C.; Deng, X., Molecular insight into the inhibition mechanism of cyrtominetin to alpha-hemolysin by molecular dynamics simulation. *Eur J Med Chem* **2013,** 62, 320-8. doi: 10.1016/j.ejmech.2013.01.008

14. Qiu, J.; Wang, D.; Zhang, Y.; Dong, J.; Wang, J.; Niu, X., Molecular modeling reveals the novel inhibition mechanism and binding mode of three natural compounds to staphylococcal alpha-hemolysin. *PLoS One* **2013,** 8, (11), e80197. doi: 10.1371/journal.pone.0080197

15. Teng, Z.; Guo, Y.; Liu, X.; Zhang, J.; Niu, X.; Yu, Q.; Deng, X.; Wang, J., Theaflavin‐3,3′‐digallate increases the antibacterial activity of β‐lactam antibiotics by inhibiting metallo‐β‐lactamase activity. *Journal of Cellular and Molecular Medicine* **2019,** 23, (10), 6955-6964. doi: 10.1111/jcmm.14580

16. Liu, S.; Zhou, Y.; Niu, X.; Wang, T.; Li, J.; Liu, Z.; Wang, J.; Tang, S.; Wang, Y.; Deng, X., Magnolol restores the activity of meropenem against NDM-1-producing Escherichia coli by inhibiting the activity of metallo-beta-lactamase. *Cell Death Discov* **2018,** 4, 28. doi: 10.1038/s41420-018-0029-6

17. Odds, F. C., Synergy, antagonism, and what the chequerboard puts between them. *Journal of Antimicrobial Chemotherapy* **2003**, (1), 1.
